# Combining microcavity size selection with Raman microscopy for the characterization of Nanoplastics in complex matrices

**DOI:** 10.1038/s41598-020-79714-z

**Published:** 2021-01-11

**Authors:** Andrea Valsesia, Monica Quarato, Jessica Ponti, Francesco Fumagalli, Douglas Gilliland, Pascal Colpo

**Affiliations:** 1 European Commission, Joint Research Centre (JRC), Ispra, Italy; 2 Present Address: International Iberian Nanotechnology Laboratory (INL), Avenida Mestre José Veiga, 4715-330 Braga, Portugal

**Keywords:** Environmental sciences, Materials science, Nanoscience and technology

## Abstract

Nanoplastic particulates (pNP) are widely considered as being potentially harmful to the environment and living organisms while also being technically difficult to detect and identify in the presence of biological matrices. In this study, we describe a method for the extraction and subsequent Raman analysis of pNP present in the tissues of salt-water mussels. The process combines a step of enzymatic digestion/filtering to eliminate the biological matrix with a detection/identification procedure, which uses a micro-machined surface, composed of arrays of cavities with well-defined sub-micron depths and diameters. This sensor surface, exploits capillary forces in a drying droplet of analyte solution to drive the self-assembly of suspended nanoparticles into the cavities leaving the individual particles isolated from each other over the surface. The resulting array, when analysed using confocal Raman microscopy, permits the size selective analysis of the individual sub-micron pNP trapped in the cavities structure.

## Introduction

The unintentional release of plastic litter into the environment is increasingly being recognized as a major threat not only for terrestrial and marine ecosystems but potentially also for human health^1^. These concerns are strongly related to their potential degradation into plastic micro and nanoparticles (pMP, pNP), both of which may exhibit size-related effects in relation to bioaccumulation and uptake levels. Such is the concern about the growing presence of particulate plastic in the environment that numerous governmental^2^ and international^3^ bodies including the European Institutions^4^, are taking action to monitor and reduce the release of plastic and microplastics^5^ which can contaminate water, soil air and even food. In recent years an increasing amount of research effort has been devoted to studying the effects of micro plastics in the environment^4^. In such studies, an important limiting factor has been applicability and reliability of established sampling protocols and the subsequent physico-chemical characterization techniques. Unfortunately, assessing the identity, quantity and size distribution of plastic that has fragmented (due to ageing, manufacturing or use) down to the sub-micron scale, even in relatively “clean” samples such as domestic water or environmental marine/fluvial water, and presents an unresolved analytical challenge. Moreover, in samples containing a significant proportion of biological material such as cells or tissue, matrix effects and residual surface contamination greatly complicate what is already a difficult analytical task.

As it is typical of emerging research fields, a single harmonized definition and classification of environmental nano, micro and meso-plastics has still to be widely agreed^6^. However, for the purposes of this study polymeric objects of anthropogenic origin, presenting colloidal behaviour and distributed in the size range between 1 and 1000 nm will be referred to as particulate nanoplastics (pNP)^7^. Similarly, particles with sizes above 1000 nm and less than 5 mm will be referred to as particulate microplastics (pMP). Analytical investigations of such systems have proven to be intrinsically complex due to a lack of reliable sampling^8^, extraction and direct identification methods^9^ which can provide quantitative information about pNP number density, size and polymer type distribution. In practice, the methods commonly used to identify microplastic particulates such as µ-Raman and µ-FTIR face intrinsic minimal size detection limitations around 1 µm micron and are unsuitable to address major questions of environmental interest such as pNP effective concentration, pNP permeation of the trophic chain and pNP impact on biological systems.

These difficulties are further compounded when dealing with samples that are rich in organic matter that must be chemically or enzymatically pre-treated to reduce the background matrix. The use of chemical digestion by oxidizing, acidic or basic media to digest biological matrices has been reported to degrade, to varying extents, even large pMP present in simulated environmental samples^10^ and it is probable that the use of such treatments on pNP or the smaller pMPs, would risk an uncontrollable loss of particulates. In such cases the softer alternative, enzymatic digestion, may provide a better compromise between digestion efficiency and sample integrity^11^, especially when dealing with samples containing a high proportion of biological matter^12,13^.

As an alternative to pre-digestion, physical separation of pNP from the bio-matrixes by hydrodynamic (i.e. field flow fractionation^14,15^ and chromatographic methods^16,17^ have been reported to give efficient recovery of primary pNP as well as good separation resolution. Unfortunately, complete removal of residual matrix material from the pNP surface remains difficult and, for all the above-mentioned techniques, stabilization of the suspension via surfactants may be required to control pNP surface charge. Surfactants and residual surface contamination may pose serious challenges for subsequent characterization.

For the detection/identification of pMPs, vibrational spectroscopic methods such as µ-FTIR and µ-Raman have been very widely reported but when applied to the smaller pNP both techniques are limited by lower size limits. For many µ-FTIR imaging systems the standard lateral resolution lies in the 10–20 µm range although this limit can be lowered down to 2 µm in more advanced set-ups (ATR objectives, FPA detectors, examining overtones of combination bands)^18–20^. µ-Raman, typically using visible monochromatic excitation, shows higher resolution then broadband IR sources-based techniques. Moreover, the diffraction limit can be physically improved by means of short-γ lasers and/or oil immersion objectives. Advanced set-ups enable the identification of single particles with diameter as small as 0.5 µm in liquid solution, when coupled with optical trapping^21^. However, laser-induced fluorescence originating from complex biomatrices surrounding the pNP or due to ageing effects may mask the characteristic pNP Raman fingerprint^22^. These interferences are recognized to be the main limitation for the Raman based detection of nanoplastics and can be addressed by photobleaching at the expense of measuring time. Unfortunately, such an approach can become impractical when scanning large surfaces (e.g. filters) or numerous particles and the development of methods, which limit matrix interferences, will be key to analyzing complex samples. Overall, an examination of the current state-of-art in the extraction analysis of nanoplastics and small (< 10 µm) microplastics reveals that there is a serious lack of straightforward, effective methods that can be used for the extraction, identification and quantification of pNP and/or small pMP in complex food and environmental samples^8,23^.

This work presents a novel approach to isolate pNP from tissue derived from biological organisms such as mussels and subsequently to enable detection on-a-chip by µ-Raman analysis. In the first step of the procedure the biological matrix is simplified by enzymatic digestion before the particulates are separated and concentrated. Next, small aliquots of the resulting liquid sample are spotted on nano-engineered surfaces composed of cavities arrays designed to entrap, by capillary force-driven self-assembly, colloidal objects according to their sizes. Once dried, a second physical simplification step is performed in which a focused ion beam (FIB) is used to preferentially eliminate biological contamination and improve the detection/identification efficiency of individual pNP via a final step of Confocal Raman Microscopy (CRM). As a proof of concept, the technique has been applied to robust pollution bio-indicators such as live mussels exposed to model polystryrene pNP dispersed in simulated sea-water^24^.

## Results

### Sensing surface and particle trapping

**Figure 1:**
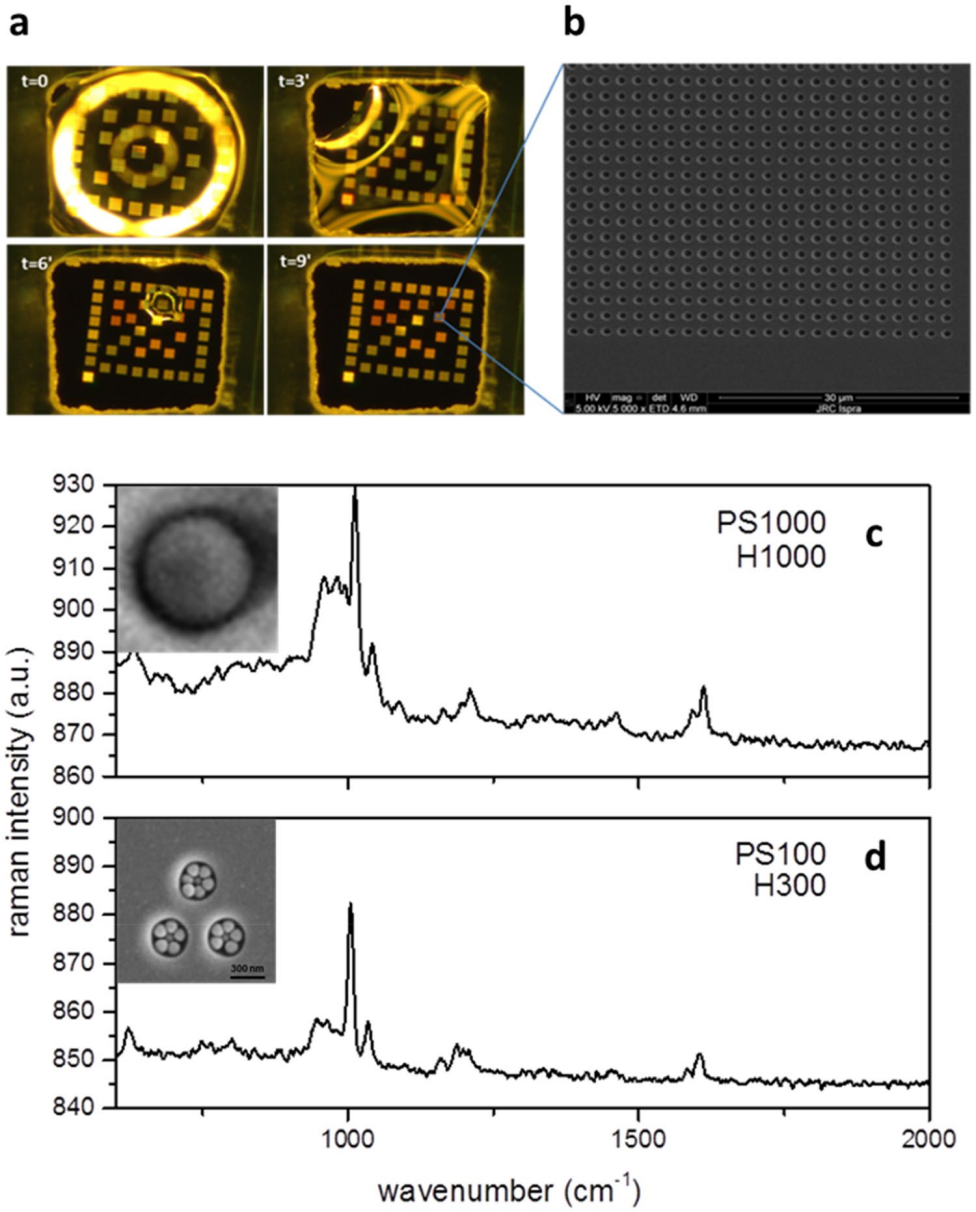
(**a**) Sequence of the drying process of the droplet inside the PDMS well and the action of capillary forces (**b**) SEM image of a portion of the array of cavities (**c**) 1000 nm PS NP trapped in a H1000 hole and relative confocal Raman spectrum (where z = 0 is the level of the optical focus) (**b**) 100 nm PS particles trapped in H300 cavity and relative confocal Raman spectrum obtained at z = − 1.45 µm (where z = 0 is the level of the optical focus).

The capability of the technique to analyze pNP in a precise size range relies on the ability of the chip to entrap nano-particles of determined size and to locate them in specific positions which facilitate their analysis by CRM. An example of the combined separation/concentration step working principle is described in Fig. 1 using solutions of 100 nm (PS100) and 1000 nm PS-NPs (PS1000) dispersed in DI-H_2_O, (without biological matrix), primary particles concentration was 9 × 10^10^ NPml^−1^. The sensing chip surface structure and its fabrication protocol is detailed in the “Methods” section. Briefly, a hydrophilic silicon wafer surface is bonded to a hydrophobic PDMS foil with pre-cut with square holes (500 × 500 µm^2^) to create micro-wells. Inside every micro-well a series of cylindrical cavities arranged in square arrays (Fig. 1a,b) are fabricated by ion milling. The arrays can have different geometrical features. In Fig. 1c,d (insets) we show two patterns: H1000, optimized for detecting particulate up to 1 µm and H300 optimized for particles up to 100 nm. A 0.5 µl droplet of analyte (here, DI-H_2_O with PS1000 and PS100) is dispensed onto the H1000 and H300 patterns and left to dry in air at room temperature. The drying process of the droplet (t = 9 min) inside the micro-well is shown in Fig. 1a. We observe a reduction of the droplet size from the periphery to the centre of the well since the patterned areas are slightly more hydrophilic than the surrounding flat silicon surface as a result of the Ga + bombardment during the milling process. Locally, the chemical contrast between patterned/un-patterned areas of the micro-wells drives the receding boundaries of the drying droplet, inducing the confinement of the particles inside the nano-holes array. In Fig. 1c (inset) one PS1000-NP trapped inside a H1000 array cavity is shown. Due to its different design, when PS100-NPs dry on a H300 array more than one particle can be confined inside the ordered cavities pattern (see Fig. 1d inset). The special design of the H300 arrays is meant to drive by self-assembly the formation of small NPs aggregates in a region comparable with the confocal volume of our CRM system (about 1 µm^3^). For similar set-up the PS single particle detection limit via CRM was found to lie around 300–500 nm (PS-pNP radius, or equivalently a volume between 0.11–0.52 µm^3^). In a group of three H300 cavities, at least 19 PS100 particles are confined (see Fig. 1d, insert) for an equivalent volume > 0.045 µm^3^, close to the single particle detection limit lower boundary, thus allowing detection via CRM (Fig. 1d). Results show how pNP self-assemble onto engineered surfaces as a function of their size thus permitting them to concentrate in a defined region compatible with the volume of the confocal analysis of our CRM system.

**Figure 2:**
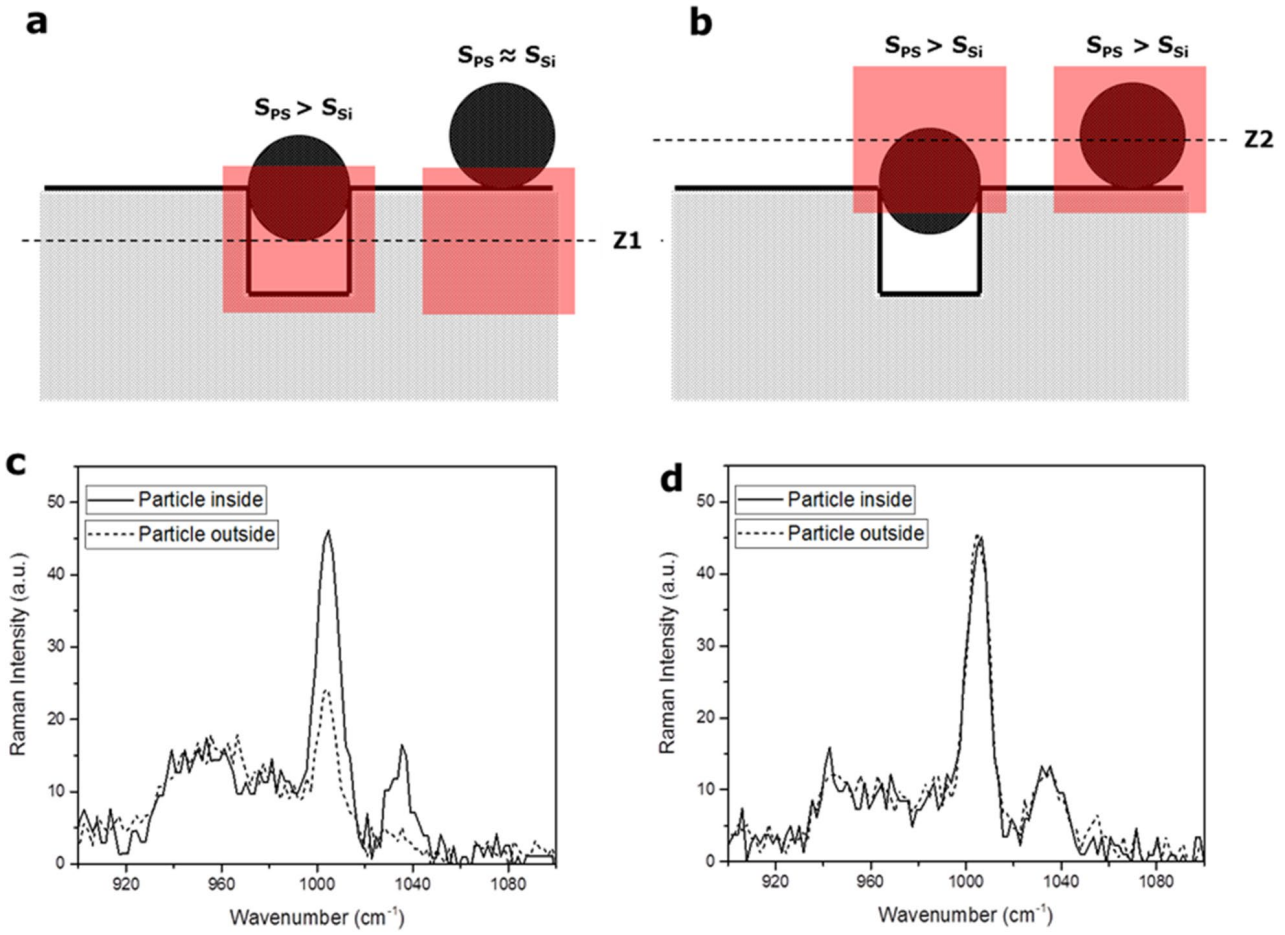
(**a**) Detection principle of the size-exclusion Raman microscopy with focus at the level underneath the silicon surface (**b**) Detection principle of the size-exclusion Raman microscopy with focus at the level above the silicon surface (**c**) Signals for the particles inside and outside the cavities at the level underneath the surface (**d**) Signals for the particles inside and outside the cavities at the level above the surface.

The CRM analysis performed on cavity arrays using DI-H_2_O spiked with PS-pNP as described in the previous section shows that it is possible to detect pNP using patterned surfaces. Moreover, using CRM, a precise depth analysis can be done by acquiring spectra at different heights with respect to the chip top surface. The working principle for detection in this manner is shown in Fig. 2. When the laser probe focal plane is placed below the Si-wafer surface, the CRM signal results from a portion of surface containing a combination of PS, air and silicon, with PS occupying the highest relative volume (for occupied cavities). In this case, the relative intensity of a polymer representative signal (i.e. aromatic ring bending vibrations, at 1005 cm^−1^)^25^ against a Si-substrate representative signal (second order scattering, at 960 cm^−1^) is representative of a pNP filled cavity. For a particle outside the cavity, the ratio of PS and Si signals decreases. On the other hand, if the focus is set above the level of the Si surface, the ratio between PS and the Si signal do not change, independently from the position of the pNP inside or outside the cavity. By scanning the nano-cavities arrays at different focal planes heights it is then possible to distinguish between pNP trapped inside the cavities and pNP remaining outside thus effectively identifying the portion of pNP below a certain size threshold.

### Exposure of mussels and subsequent enzymatic digestion

**Figure 3:**
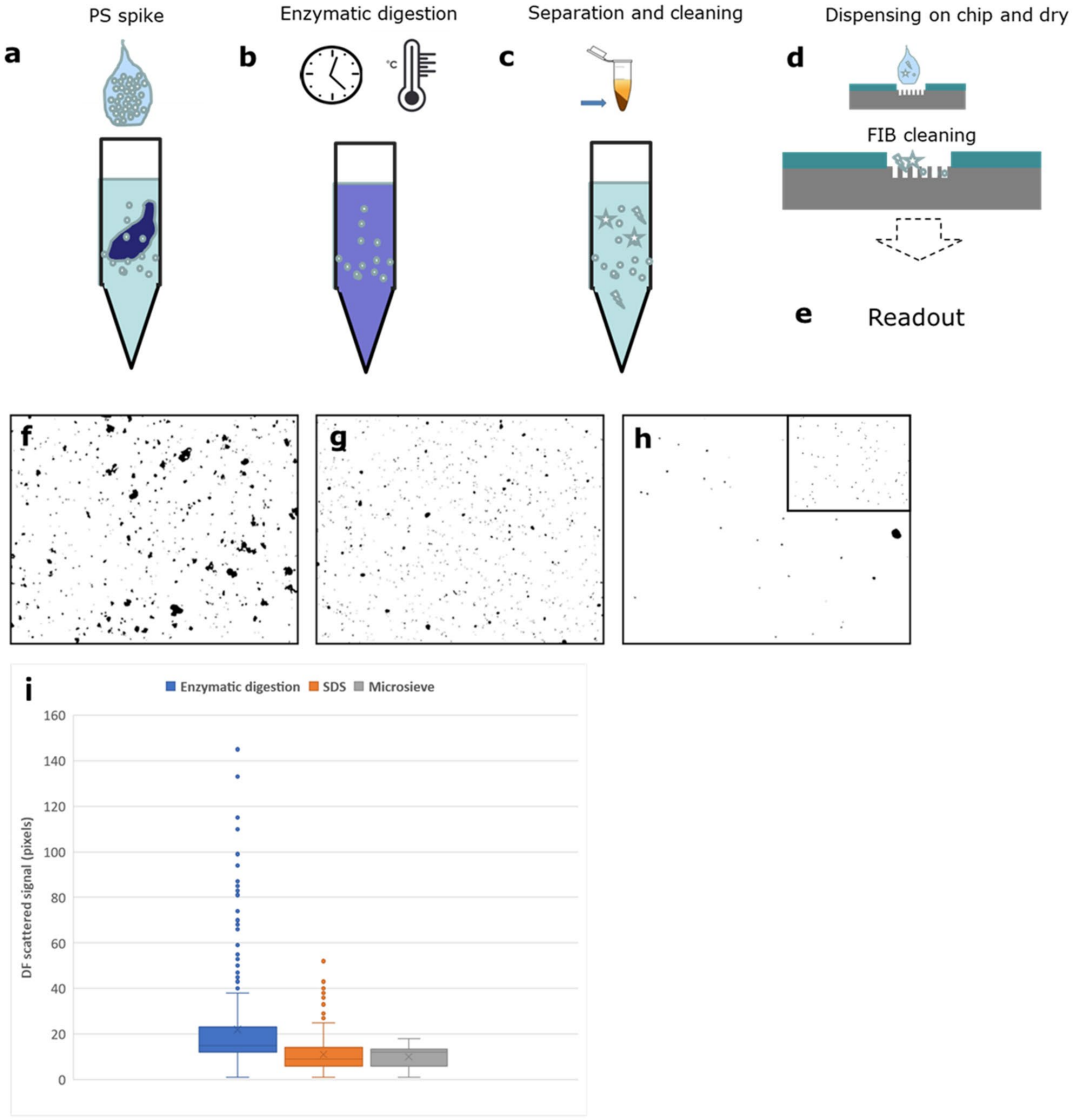
Schematics of the preparation method (**a**) spiking of the entire animal with nanoparticles (**b**) enzymatic digestion (**c**) separation and cleaning of the matrix (**d**)–(**e**) dispensing on the chip, FIB cleaning and readout (described in the next paragraphs). (**f**)–(**h**) Objects recognized by dark-field microscopy of samples after enzymatic digestion, after adding SDS and after filtering with micro-sieves. In the inset in (**h**) a typical dark-field signal from PS beads of 500 nm is shown. (**i**) distribution of the scattering signal of the objects by DF after each step of the sludge purification.

After establishing the basic methodology using a simple pNP-H_2_O solution, the analysis procedure was further developed and tested for use with samples from saltwater mussels exposed to pNP as an example of an aquatic species commonly consumed by humans. The four main operational steps applied to the mussels after exposure to pNP-H_2_O were (1) enzymatic digestion of biological tissue and biomatrix in solution, (2) centrifugation and re-dispersion in water (3) self-assembly on-a-chip and physical treatment by ion beam to further reduce the background matrix on the surface, (4) detection by Raman Confocal Microscopy. The method steps are visualized in Fig. 3a–e.

**Figure 4:**
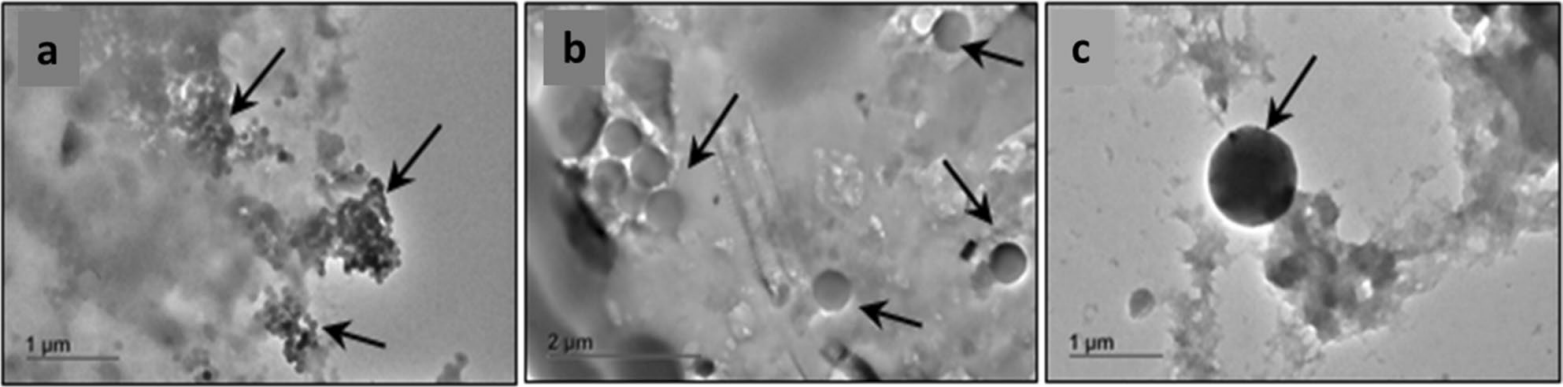
TEM images of the pNP recovered with the method described in this paper from the mussels’ experiments for (**a**) 100 nm PS pNP (**b**) 500 nm PS pNP (**c**) 1000 nm PS pNP.

In order to produce the starting test materials, live mussels were exposed for 24 h to simulated seawater containing controlled amounts of pNP (PS). A relatively large concentration of different PS-pNP was added to the 200 ml of seawater. The resulting solution concentrations were 4.55 × 10^8^ NP/mL (1 µm), 3.64 × 10^9^ NP/mL (0.5 µm) and 4.55 × 10^11^ NP/mL (0.1 µm). Exposure experiments, tissue digestion and pNP recovery are shown in Fig. SI1. After exposure the mussels were removed from the seawater and rinsed with MilliQ (MQ) water. For each exposure time point one whole animal was completely homogenized using a proteolytic enzyme (Papain, see “Methods” section). After enzymatic digestion the biomatrix protein-components were found to be degraded leaving only a viscous suspension mainly composed of residual lipid components, particulate debris (pNP and other, mainly inorganic, components such as micro-plankton) and sand. The suspended residuals were then solubilized by adding sodium dodecyl sulphate (SDS, 10%vol.). In this step the SDS plays a dual role: it dissociates the non-covalent bonds of residual protein fragments and acts as surfactant to disperse lipids and fatty acids to form a transparent, non-viscous solution (see SI and Fig. S1e). The solution was then centrifuged, and the pellet re-dispersed in DI-H_2_O (1 ml), sub-sampled (0.1 ml), and again diluted 1:100 vol./vol. in MQ water before filtration through clean Si_3_N_4_ microsieves with 2 µm pore size. The sample was then centrifuged again, and the pellet obtained dispersed in MQ water (0.3 ml). The concentration of objects (including particulates) with diameter less than 1 µm for different steps of sample preparation is shown in Table SI1. To determine concentrations, for each step, 5 µl of concentrated sample were spotted onto glass slides and covered with a coverslip. Dark field (DF) optical microscopy (10 × objective) and image analysis software were then used to measure the size distribution of particulates in the samples. Figure 3f–h show the DF microscopy images and measured particle scattering distributions after sludge separation, pellet purification. Figure 3h (inset) shows the reference distribution obtained by spiking ultrapure water with PS-pNP (1 µm). Figure 3i shows the distribution of the scattering signal of the objects by DF after each step of the sludge purification, being the scattering signal proportional to the size. The size of the objects thus decreases after each step of the process, with a particular decrease between the enzymatic digestion and the SDS adding process. After purification, the resulting complex homogenate matrix exhibits viscosity and density suitable for homogenous drop-cast deposition as a thin film on the chip surface. The TEM images of samples extracted using the previously described method are shown in Fig. 4. The pNP size is not affected by enzymatic digestion and the subsequent cleaning steps.

### Focused Ion Beam selective cleaning of the biological matrix.

**Figure 5:**
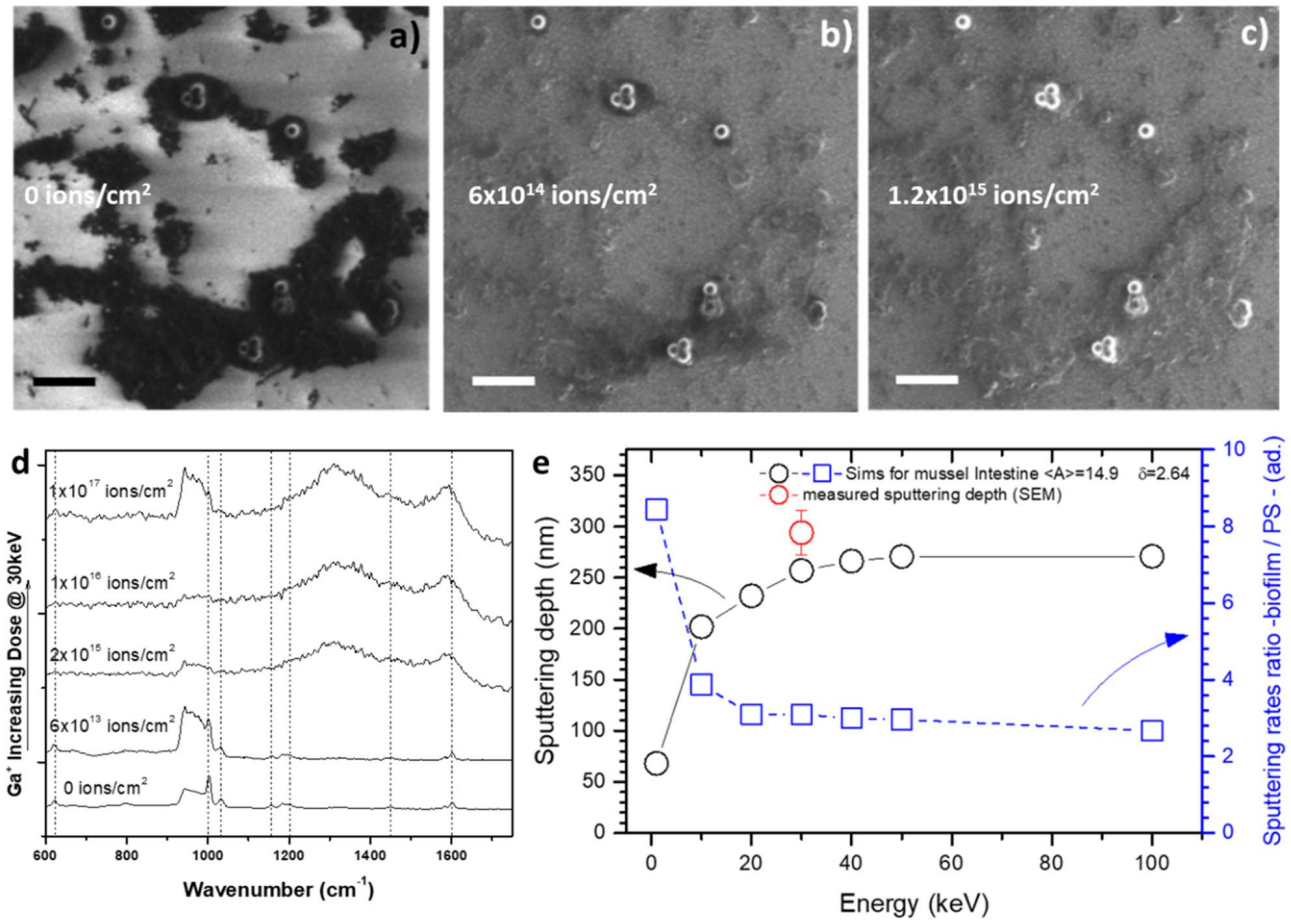
(**a**) SEM image of mussel matrix and the PS-NP casted on a silicon chip (**b**) same image of (**a**) with a dose of 6 × 10^14^ Ga^+^ ions (**c**) same image of (**a**) with a dose of 1.2 × 10^15^ Ga^+^ ions. (**d**) Evolution of the confocal Raman spectra as a function of the Ga^+^ ions dose (**e**) SRIM calculation of the sputtering depth for the mussel matrix and of the sputtering ratio between mussel matrix and the pNP as a function of the acceleration voltage of the ions. Scale Bar is 500 nm.

Enzymatic digestion, centrifugation and filtering are effective in eliminating a large part of the biological matrix from the pNP. However, SEM analysis of the nano-cavities arrays surfaces spotted with filtered sample show that, at the nanoscale, a non-negligible portion of the biomatrix continues to surround the candidate nanoparticles objects, hindering their spectroscopic identification. To address this issue, FIB treatment may be applied to the surface to completely remove the organic matrix. A purely ballistic approach was preferred over technically simpler methods involving oxidative chemical etching (e.g. oxygen plasma etching or UV exposure) in order to avoid: (1) the formation of an oxidation layer within the pNP and (2) the formation of a residual, hard to etch, amorphous-carbon coating^26^ that may hamper their subsequent spectroscopic identification. The effect of FIB exposure on the biomatrix/pNP sample cast on a silicon chip is shown in Fig. 5a–c, as a function of the ion dose. Ga^+^ irradiation selectively removes the biomatrix, while leaving the pNP virtually intact. Moreover, a sharp increase in the contrast between biomatrix-covered areas and pNP can be observed already at a dose of 6 × 10^14^ ions/cm^2^ (Fig. 5b). Surface imaging as a function of ion dose suggests a process in which, under ion beam exposure, the biological matrix is being selectively etched and removed from the substrate while different nano-sized objects with a different chemical composition (mean atomic number) and densities remain relatively unaffected. We can explain these different sputtering rates by considering the relative sputtering yields between the candidate pNP objects and the biomatrix. The sputtering yields were calculated using SRIM simulations (see “Methods” section), as a function of the ion beam energy. The results are shown in Fig. 5e. For the case of FIB experiments in the energy range of 0–100 keV, the process is well represented by linear cascade sputtering regime approximation^27^ where the yield is a function of the beam energy (ε), the atomic weight ratio between incident ions and target atoms (M_Ga+_/M_target_), material atomic composition and material density (δ). A density value of 1.05 g cm^−3^ for pNP was used (bulk reference value of PS) while for the biofilm a value of 2.69 g cm^−3^ was measured (see SI). The corresponding atomic compositions were derived from EDX experiments (see Table SI2). The cast sample can be approximated as being a heterogeneous multicomponent target (pNP and biofilms) within which the sputtering rate induced by Ga + ions of each domain can be easily calculated from the sputtering theory^27^ (see Fig. 5e and SI). The sputtering depth for a dose of 2.9 × 10^17^ ions cm^−2^ has been calculated and plotted in Fig. 5e and verified using SEM images of sputtered biomatrix films cross sections and isolated pNP. The results show that at about 30 keV the sputtering yields for the investigated target materials, reaches about 90% of its high-energy saturation value. Consequently, in order to etch a depth of material equivalent to the size of the particles using a reasonable dose, we choose an acceleration voltage of around 30 kV. The FIB process selectivity can be evaluated using the etching ratio (ER_Biofilm/PS_) between the biomatrix and the pNP. Figure 5e shows that ER_Biofilm/PS_ at 30 keV, where the sputtering rate for the biomatrix is more efficient, is close to 3. In order to check for possible modifications of the PS structure induced by Ga^+^ (30 keV, different doses) bombardment, CRM was performed on isolated pNP on Si-wafer by spin-coating. The resulting spectra are shown in Fig. 5d. The SEM image of the irradiated areas are shown in Figure SI2. In the case of the samples treated at the three higher doses, the heat generated by ion impact has the effect of raising the pNP temperature above *T*_*g*_, creating a new phase with amorphous carbon character as evidenced by the broad peaks at 1300 and 1600 cm^−1^. In contrast, for doses lower than 2 × 10^15^ ions/cm^2^, the typical vibrations of the polymer remain visible at their normal spectral positions. For example, the typical aromatic ring bending vibration (at 1005 cm^−1^) is detectable with a dose of 6 × 10^13^ ions/cm^2^.

### On-a-chip pNP identification in complex matrix

**Figure 6:**
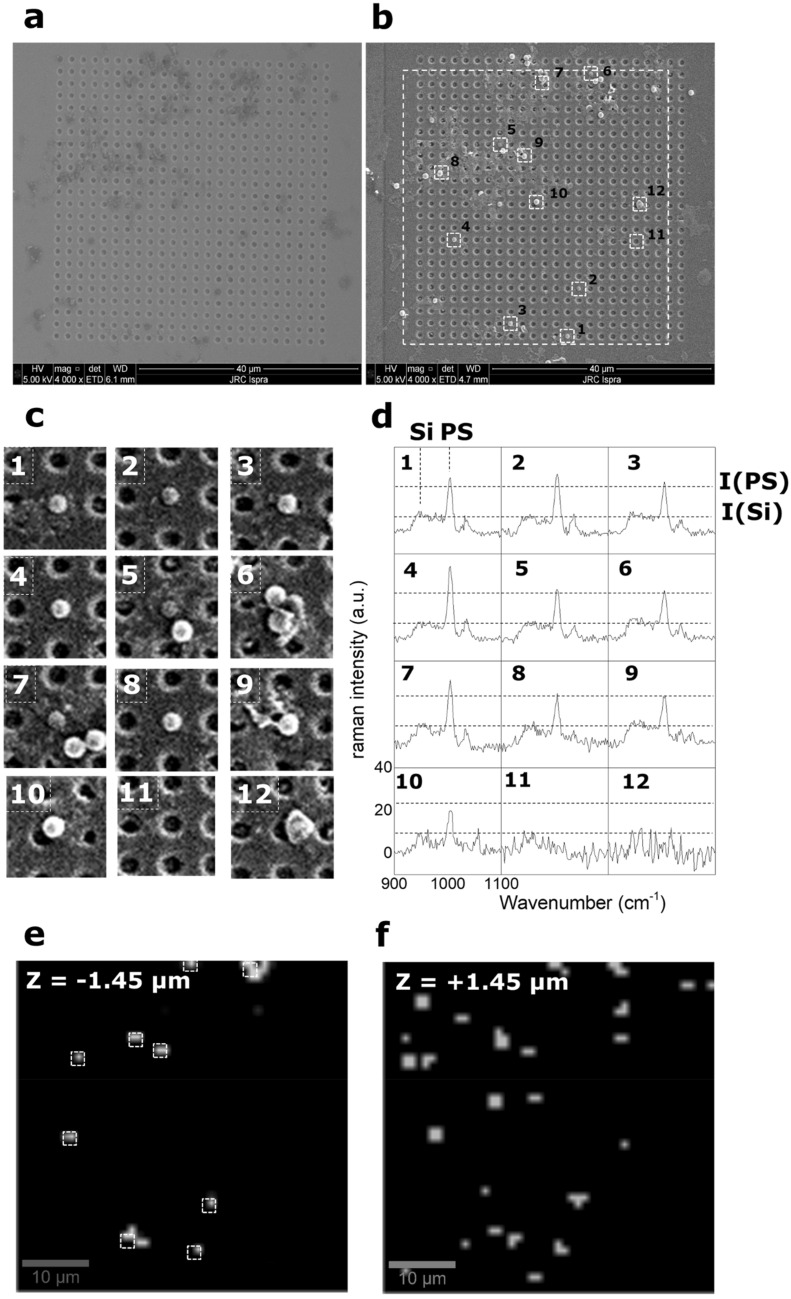
(**a**) SEM image of the array of holes after drying of the sample containing the digested mussel matrix and the pNP. (**b**) SEM image of the array of holes after drying of the sample containing the digested mussel matrix and the pNP and after the selective cleaning of the surface by Ga^+^ FIB (**c**) zoom of the squares indicated in (**b**) corresponding to the areas where pNP were identified by SEM analysis. (**d**) Corresponding Raman spectra of the areas in (**c**) acquired at z = − 1 µm. The secondary peak for Silicon and the peak corresponding to the aromatic ring for PS are indicated as well as their Raman intensity. For particles identified with the numbers 1 to 9 the intensity of the PS aromatic ring is > than the double of the one for the secondary Si peak. For objects identified with number from 10 to 12, the intensity of the PS aromatic ring is < than the double of the one for the secondary Si peak (as in the case of object 10) or the peak are not present (objects 11 and 12). (**e**) 3-D hyperspectral image acquired at z = −1.45 µm and I(PS) > 2*I(Si). (**f**) 3-D hyperspectral image acquired at z = +1.45 µm and I(PS) > 2*I(Si).

The four processes previously described have then been used to identify and characterize PS1000 pNP on-a-chip, starting from tissue which had been recovered from live mussels exposed to artificial seawater doped with the PS particles. Droplets (500 nL) of the digested and filtered biomatrix were dispensed inside the PDMS well containing several size-exclusion arrays of micro-cavities. At this stage, to increase the amount of analyte on the collector surface, additional droplets (up to 5 µl) were sequentially spotted and dried inside the well. Prior to the FIB processing, only the residual biomatrix material can be seen covering the surface (Fig. 6a), while after ion beam exposure (30 kV and 6 × 10^13^ ions cm^−2^) several pNP candidates objects become clearly visible (Fig. 6b). Based on the nominal concentration of PS pNP at the end of the separation and concentration steps (1.51 × 10^6^ NP ml^−1^), 5 µl of analyte would be expected to contain approximatively 7550 particles. Assuming a homogenous particles distribution inside the well, the number of particles per cavity array in our configuration should be: N = 7550*(1600 µm^2^)/(640,000 µm^2^) ~ 19 particles. If we consider the collector shown in Fig. 6a,b total of 23 particles were found on the 40 × 40 µm^2^ array area supporting the conclusion that there are no significant losses during the digestion, separation and simplification processes. Moreover, the use of micro-cavities array facilitates the localized concentration of particles due to the capillary forces during the drying process. Different particulate objects were found on the array, in some cases there are single particles without a surrounding matrix perfectly fitting inside the holes while in others there are particles aggregated with residual surrounding matrix and other bigger particles. In particular, 10 particles were recognized to be fitting inside the holes or standing in proximity of them (white squares in Fig. 6b and Fig. 6c). The holes-covered area was scanned by Confocal Raman Microscopy (CRM) in order to characterize the objects trapped inside the holes of the array. As previously described, the discrimination between particles inside and outside the cavities was done by focusing the microscope at height of −1.45 µm with respect to the focal plane of the image, denominated z = 0 µm, which corresponds with the surface of the un-milled silicon. In this way, 4-Dimensional hyperspectral data are created. The spectra obtained in the areas indicated in Fig. 6c with numbers from 1 to 12 are shown in Fig. 6d. The spectra corresponding to objects 1 to 10 in the selected spectral range show the typical Silicon peak at 960 cm^−1^ and the aromatic ring vibration of the PS at 1005 cm^−1^. The intensity of the two peaks with respect to the baseline are indicated respectively with *I(Si)* and *I(PS)*. For objects 1 to 9, *I(PS)* > 2*I(Si) indicating that these particles lie inside the cavity or in near proximity of it. In case of object 10, *I(PS)* ~ *I(Si)* indicating that the particle lies outside the cavity, as also shown in the corresponding SEM image. A spectrum for an “empty” hole (object number 11) or a non-identified object (number 12) are shown as a mean of comparison. Analysis by band integration provides 3-D intensity map of the hyperspectral maps. The integration was performed for PS (1000–1005 cm^−1^) and silicon (950–960 cm^−1^) characteristic signals. The map was scaled in a way that the colour is white for *I(PS)* > 2*I(Si) and black for any other value. The map acquired at z = −1.45 µm clearly shows the 9 objects inside the holes as white areas (Fig. 6e). On the other hand, using the same criteria, many more objects are identified as white areas for the hyperspectral map (reduced by univariate analysis) acquired at z =  + 1.45 µm as shown in Fig. 6f.

## Discussion

At the time of this writing, review papers^28^ cite only two published methodologies for pNP detection, both designed for environmental matrices^29^. In contrast, many reports are found about pMP detection, not only in water but also in more complex media such as food, fish and shellfish^30^. The reason for this disparity is mainly attributable to the lack of reliable methods for pNP extraction and purification from complex media, as evidenced by other authors^30^. While the use of enzymatic digestion by Papain has been shown to be effective it is not sufficient to achieve a complete removal and separation of pNP from bio-matrices. This occurs because no single enzyme is equally effective at removing each of the different components of the complex biomatrix (lipids, proteins, nucleic acids, organelles, etc.). While it is possible that this limitation could be reduced by using a sequence of different enzymes or possibly a single cocktail of several enzymes, these approaches greatly increase protocol complexity, cost and treatment time. Furthermore, such an approach may lead to the formation of a hard protein corona around pNP, as already demonstrated in literature^31^. The formation of a hard corona implies a strong bonding of the biological matrix to the particles surface, as we observe before the ion beam processing, as well as agglomeration and bridging between particles with consequent modification of the protein structure. In this study, the combination of enzymatic and physical steps to reduce the organic matrix enables almost complete separation and purification of pNP from the biological material. Finally, the FIB surface treatment shows selectivity for any biological matrices which exhibits density and chemical composition different from common polymers, as verified via SRIM calculation. We need to point out that after the enzymatic digestion, even if not removed completely, the biomatrix is already altered in terms of its density. This is demonstrated by the fact that an extremely low flux of ions (6 × 10^13^ ion cm^−2^) enables removal of the matrix residues without affecting the structure of the nanoplastics as shown by the Raman spectra (Fig. 6d). The combination of enzymatic digestion and ion beam sputtering results therefore in a successful separation of pNP from the biological matrix. Moreover, the combination of the FIB cleaning and the electron imaging in the same instrument (as in the dual-beam electron microscopes) enables a quick identification of nano-objects by their elemental composition. A rapid qualitative discrimination between polymers, metals and inorganic particles could be performed using electron imaging combined by in situ elemental analysis by Energy-dispersive X-ray spectroscopy (EDS, a technique that is often present in standards SEM microscopes. EDS can rapidly reveal which elements make up the bulk of each nanoparticle with a resolution of few nm. As an example, we report in Figure SI4e the EDS elemental analysis of nano-objects detected by SEM after the FIB cleaning (Figure SI4a-c). The electron imaging reveals the presence of regular, spherical objects with dimensions of 60–80 nm (Figure SI3d) which, from their regular morphology, could potentially be manufactured pNP. However, the elemental analysis reveals that these objects are composed mainly of Ca and O, indicating that they are minerals naturally produced inside the mussel, excluding the possibility of them being anthropogenic nanopolymers. This example is contrast to what would be expected with a true pNP which would be composed principally of elements like C, O, N, Cl possibly with minor quantities of other elements attributable to additives or contaminants.

More importantly, Raman spectroscopy enables the chemical identification of the particles when they are distributed and separated by the nano-cavities array surface, enabling also the discrimination of the particles according to their size. We would like to stress the fact that the correlation with SEM and FIB cleaning is crucial for understanding the nature of the nanoparticles fitting inside the cavities. It is possible in fact to discriminate between single particles or particle aggregates as shown in Fig. 1b. In case of particles heteroaggregates, it is possible in principle to spectrally resolved the different components using advanced chemometrics techniques^32^. The detection and identification of the different components in heteroaggregates represent an interesting follow up of the present research.

## Conclusion

In this work a novel method is described for the detection and sizing of pNP extracted from complex biological matrixes such a whole mussel. The overall process combines a step of enzymatic digestion/filtering to eliminate the organic matrix with a detection/identification procedure that uses a micro-machined surface as an aid to particle detection and sizing by Confocal Raman microscopy. An intermediate step of cleaning of the matrix by Focused Ion Beam greatly improves the possible the detecting and identifying the pNP. As a proof of concept, the methodology has been applied to samples made by deliberate spiking of mussels’ tissue as well as to whole mussels that have taken up particulates during live exposure in artificial seawater. The method described here shows great potential for the analysis of pNP in complex organic matrices and shows how a combination of different analytical and microscopic techniques makes their detection and identification possible. Furthermore, by exploiting a novel mechanism of size exclusion that traps individual particles smaller than a certain size the method provides a means to collect, concentrate and detect particles in an analysis volume that is smaller than that which is normally possible with the resolution and sensitivity limitations of standard Raman microscopes.

## Methods

### Enzymatic digestion

To provide nanoparticle samples for method development commercially grown mussels were purchased from local supermarket and stored at − 20 °C after shell removal. Prior to use the mussels were defrosted, weighed and spiked with 1 µl pNP of 1 µm in size (9 * 10^10^ NPs/ml). To perform the enzymatic digestion Papain from Carica papaya has been used. Papain is a proteolytic enzyme that belongs to the cysteine proteinase family best known for its tenderising properties. Prior to use the papain was mixed with an activation buffer (0.5 mg/ml) consisting of 1.1 mM EDTA, 0.067 mM mercaptoethanol and 5.5 mM cysteine-HCl and incubated at 30 °C for 30 min. Once activated, the enzyme solution at a concentration of 0.5 mg/ml. was mixed with about 0.5 g of the sample of spiked mussel and the digestion allowed to proceed overnight at 40 °C. After digestion the residue was centrifuged (1500 rcf, 15 min) and dispersed in ultrapure water. Sodium Dodecyl Sulphate (SDS, Sigma) was then added to the solution before being diluted in ultrapure water and filtered using a high-porosity microsieve chip (Aquamarijn Micro Filtration BV) with 2 µm pores. The filtered solution was collected and used for the analysis. The solution was then stored at + 4 °C before SEM analysis.

### Surface nanofabrication and capillary drying

A SEM instrument equipped with a Focused Ion beam (Nova 600i, Nanolab, Thermofisher, Eindhoven, The Netherlands) was used to create arrays of cavities for later use in analysis by size-exclusion Raman. To produce the arrays a standard Silicon chip (p-doped, resistivity < 1 Ω) was milled by the FIB at an acceleration voltage of 30 keV and aperture current of 2.8 nA. The pattern etched into the surface of the silicon was controlled by the instrument using a bitmap image file generated as an electronic template. The standard image file consisted of a square array of 40 × 40 white dots of diameter D, separated from each other by a distance L = D . Two different patterns have been prepared: holes with 1.25 µm separated by 2 µm (H1000) and three holes of 300 nm size positioned on the vertexes of an equilateral triangle with side of 1 µm (H300). During the nanofabrication, the acceleration voltage and the current (beam size) of the ion beam were optimized in order to avoid re-deposition of the sputtered silicon and cross-irradiation effects. To induce the controlled drying of the liquid samples to the patterned silicon surface the array of holes were fabricated inside a fluidic well made by placing a thin PDMS foil with a pre-cut square hole onto the silicon substrate. In order to cover a greater part of the exposed silicon area several arrays were fabricated inside the well. In order to ensure the correct alignment of the arrays of holes within the well, the PDMS foil is firstly fixed on the unmodified silicon chip and then inserted in the SEM chamber. The electron imaging is used to align the Ion Beam to produce the holes in the desired areas. In order to avoid distortion of the ion beam due to the presence of the insulating PDMS (which charges upon the ion beam irradiation), the arrays of holes were fabricated at distance of at least 50 µm from the walls of the PDMS well. It was noted that a side effects of ion beam irradiation of the silicon surface was to locally increase of the hydrophilicity (decrease of the contact angle with water) of the surface. Droplets of 500 nL of sample were placed in the well where they were found to dry within a few seconds. The drying process was observed by an optical microscope. Scanning Electron Microscopy was done on the dried particles using the Nova 600i Nanolab by detecting the secondary electrons at 5 kV acceleration voltage and different apertures.

### Scanning electron microscopy, energy dispersive spectroscopy and focused ion beam differential sputtering

SEM, EDS and FIB were carried out in a Nova 600i Nanolab (Thermofisher, Eindhoven, The Netherlands) equipped with a EDS system for elemental analysis (EDAX Inc, Mahwah, NJ, USA).

For the FIB, the ion current used was 1.5, 28, 280 and 2800 pA with acceleration voltage of 30 kV, the dose was controlled by the time of scanning of the ion beam on the selected area. The differential sputtering calculation has been performed by SRIM software (version SRIM-2013 http://srim.org/index.htm). The mussel’s homogenate density was measured by combining quartz crystal microbalance (QCM) (Q-Sense, Biolin Scientific, Gothenburg, Sweden) and profilometry (Alphastep, KLA Tencor, Milpitas, California, USA). A volume of 20 µl of digested homogenate was spin-coated at 3000 rpm for 2 min (Laurell Technolgies, North Wales, PA, USA) on a QCM standard quartz. In this way a homogenous film is created. The resonance frequency of the quartz has been previously measured. Then the frequency shift is measured after the deposition of the film and the mass is derived by the Sauerbrey equation. A profilometric measurements of the thickness of the film enables the calculation of the volume of the deposited homogenate disk on the QCM crystal. The calculation of the density has been done by dividing the mass by the volume, yielding a value of  2.7 ± 0.2 g cm^−3^ where the main source of error is introduced by the profilometric thickness measurements.

### Confocal Raman microscopy

Witec confocal microscope (Witec, Ulm, Germany) equipped with red laser light (633 nm) was used to scan the array of holes. The scan resolution was 200 nm and the integration time per pixel was 5 s. A 100× magnification object was used with numerical aperture of 1.25. Univariate analysis of the hyperspectral images was performed using the Witec instrument software (Witec Suite 5 https://www.witec.de/products/accessories/software-witec-suite/) by integration of the spectra corresponding to the spectral bands of the Si and of the benzene ring vibration of the PS. The colour scale image is calculated as follows:

$${\text{I}}\left( {{\text{x}},{\text{y}}} \right) \, = {\text{ I}}\left( {{\text{x}},{\text{y}},{\text{z}} = {\text{z}}_{0} , \, \Delta {\text{I}}\left( {\uplambda_{{{\text{PS}}}} } \right)/ \, \Delta \uplambda_{{{\text{PS}}}} } \right) \, {-}{\text{ I}}\left( {{\text{x}},{\text{y}},{\text{z}} = {\text{z}}_{0} , \, \Delta {\text{I}}\left( {\uplambda_{{{\text{Si}}}} } \right)/\Delta \uplambda_{{{\text{Si}}}} } \right)$$

where Δλ_PS_ = 1005–1000 cm^−1^ and Δλ_Si_ = 960–950 cm^−1^.

Cosmic Rays Removal and baseline correction tools were applied to the spectra before the univariate analysis.

### Exposure of live Mussels

Live, commercially available, farmed (Goro, Ferrara, Italy) mussels (Mytilus Galloprovincialis), were sourced from a local supermarket. Three fresh mussels were exposed to a mixture of polystyrene beads of 1, 0.5 and 0.1 µm diameter size (Polybead Microspheres, Polysciences, UK) for 24 h in 200 mL synthetic seawater (ASTM D1141-98, pH 7.5–8.4, Sigma Aldrich) with magnetic stirring at 15 °C. Negative control (unexposed mussels) was also included and used to obtain material for experiments in which polystyrene beads were directly added to organic matrix. The matrix after the digestion were analysed by Transmission Electron Microscopy, TEM, at 120 kV (JEOL-JEM2100, JEOL, France).

## Supplementary information

Supplementary Information 1.

## References

[1] Vethaak AD, Leslie HA (2016). Plastic debris is a human health issue. Environ. Sci. Technol..

[2] Kentin E, Kaarto H (2018). An EU ban on microplastics in cosmetic products and the right to regulate. Rev. Eur. Comp. Int. Environ. Law.

[3] United Nations Environmenal Programme. Marine Litter: A Global Challenge. in *Marine Litter : A Global Challenge* (ed. United Nations).

[4] Nyka M (2019). Legal approaches to the problem of pollution of marine environment with plastic. Sci. J. Marit. Univ. Szczecin.

[5] Haines A, Scheelbeek P (2020). European Green Deal: A major opportunity for health improvement. Lancet.

[6] Hartmann NB (2019). Are we speaking the same language? Recommendations for a definition and categorization framework for plastic debris. Environ. Sci. Technol..

[7] Gigault J (2018). Current opinion: What is a nanoplastic?. Environ. Pollut..

[8] Schwaferts C, Niessner R, Elsner M, Ivleva NP (2019). Methods for the analysis of submicrometer-and nanoplastic particles in the environment. TrAC Trends Anal. Chem..

[9] Shim WJ, Hong SH, Eo SE (2017). Identification methods in microplastic analysis: A review. Anal. Methods.

[10] Hurley RR, Lusher AL, Olsen M, Nizzetto L (2018). Validation of a method for extracting microplastics from complex, organic-rich, environmental matrices. Environ. Sci. Technol..

[11] Courtene-Jones W, Quinn B, Murphy F, Gary SF, Narayanaswamy BE (2017). Optimisation of enzymatic digestion and validation of specimen preservation methods for the analysis of ingested microplastics. Anal. Methods.

[12] Bouwmeester H, Hollman PCH, Peters RJB (2015). Potential health impact of environmentally released micro-and nanoplastics in the human food production chain: Experiences from nanotoxicology. Environ. Sci. Technol..

[13] Catarino AI, Macchia V, Sanderson WG, Thompson RC, Henry TB (2018). Low levels of microplastics (MP) in wild mussels indicate that MP ingestion by humans is minimal compared to exposure via household fibres fallout during a meal. Environ. Pollut..

[14] Gigault J, El Hadri H, Reynaud S, Deniau E, Grassl B (2017). Asymmetrical flow field flow fractionation methods to characterize submicron particles: Application to carbon-based aggregates and nanoplastics. Anal. Bioanal. Chem..

[15] Correia M, Loeschner K (2018). Detection of nanoplastics in food by asymmetric flow field-flow fractionation coupled to multi-angle light scattering: Possibilities, challenges and analytical limitations. Anal. Bioanal. Chem..

[16] Tiede K (2009). A robust size-characterisation methodology for studying nanoparticle behaviour in ‘real’environmental samples, using hydrodynamic chromatography coupled to ICP-MS. J. Anal. At. Spectrom..

[17] Gray EP (2012). Analysis of gold nanoparticle mixtures: A comparison of hydrodynamic chromatography (HDC) and asymmetrical flow field-flow fractionation (AF4) coupled to ICP-MS. J. Anal. At. Spectrom..

[18] Prata JC, da Costa JP, Lopes I, Duarte AC, Rocha-Santos T (2019). Effects of microplastics on microalgae populations: A critical review. Sci. Total Environ..

[19] Lenz R, Enders K, Nielsen TG (2016). Microplastic exposure studies should be environmentally realistic. Proc. Natl. Acad. Sci..

[20] Mintenig SM, Löder MGJ, Primpke S, Gerdts G (2019). Low numbers of microplastics detected in drinking water from ground water sources. Sci. Total Environ..

[21] Gillibert R (2019). Raman tweezers for small microplastics and nanoplastics identification in seawater. Environ. Sci. Technol..

[22] Käppler A (2016). Analysis of environmental microplastics by vibrational microspectroscopy: FTIR, Raman or both?. Anal. Bioanal. Chem..

[23] Alexy P (2020). Managing the analytical challenges related to micro-and nanoplastics in the environment and food: Filling the knowledge gaps. Food Addit. Contam. Part A.

[24] Li J (2019). Using mussel as a global bioindicator of coastal microplastic pollution. Environ. Pollut..

[25] Yabagi, J. A. *et al.* The effect of gamma irradiation on chemical, morphology and optical properties of polystyrene nanosphere at various exposure time. In *IOP Conference Series: Materials Science and Engineering* vol. 298 (2018).

[26] Fumagalli F, Hanuš J, Kylián O, Rossi F (2012). In situ quartz crystal microbalance measurements of thin protein film plasma removal. Plasma Process. Polym..

[27] Nastasi M, Mayer JW, Wang Y (2014). Ion Beam Analysis: Fundamentals and Applications.

[28] Lehner R, Weder C, Petri-Fink A, Rothen-Rutishauser B (2019). Emergence of nanoplastic in the environment and possible impact on human health. Environ. Sci. Technol..

[29] Ter Halle A (2017). Nanoplastic in the North Atlantic subtropical gyre. Environ. Sci. Technol..

[30] Nguyen B (2019). Separation and analysis of microplastics and nanoplastics in complex environmental samples. Acc. Chem. Res..

[31] Gopinath PM (2019). Assessment on interactive prospectives of nanoplastics with plasma proteins and the toxicological impacts of virgin, coronated and environmentally released-nanoplastics. Sci. Rep..

[32] Camp CH (2019). pyMCR: A python library for multivariate curve resolution analysis. J. Res. (NIST JRES).

