# Supplementary material for: Combining microcavity size selection with Raman microscopy for the characterization of Nanoplastics in complex matrices
Source: Sci Rep. 2021 Jan 11;11:362. doi: 10.1038/s41598-020-79714-z (PMC7801455; doi:10.1038/s41598-020-79714-z)
Supplement: Supplementary file 1 — Supplementary Information 1. [file 41598_2020_79714_MOESM1_ESM.pdf]

Supporting Information

Combining microcavity size selection with Raman microscopy for the characterization of Nanoplastics in complex matrices

Andrea Valsesia, Monica Quarato, Jessica Ponti, Francesco Fumagalli, Douglas Gilliland and Pascal Colpo

European Commission, Joint Research Centre (JRC), Ispra, Italy,

Table SI1. Nominal estimated concentration of PS NPs of diameter of 1 µm in the different steps of the separation process

**Step**	**Description**	**Concentration (NP/ml)**
1	Spike of the mussel	$4.55x10^{7}$
2	Enzymatic digestion	$4.55x10^{7}$
3	SDS cleaning and first centrifugation	$4.55x10^{7}$
4	Dilution for filtering	$4.55x10^{5}$
5	Centrifugation and re-dispersion in a 10 times smaller volume	$1.51\ x10^{6}$

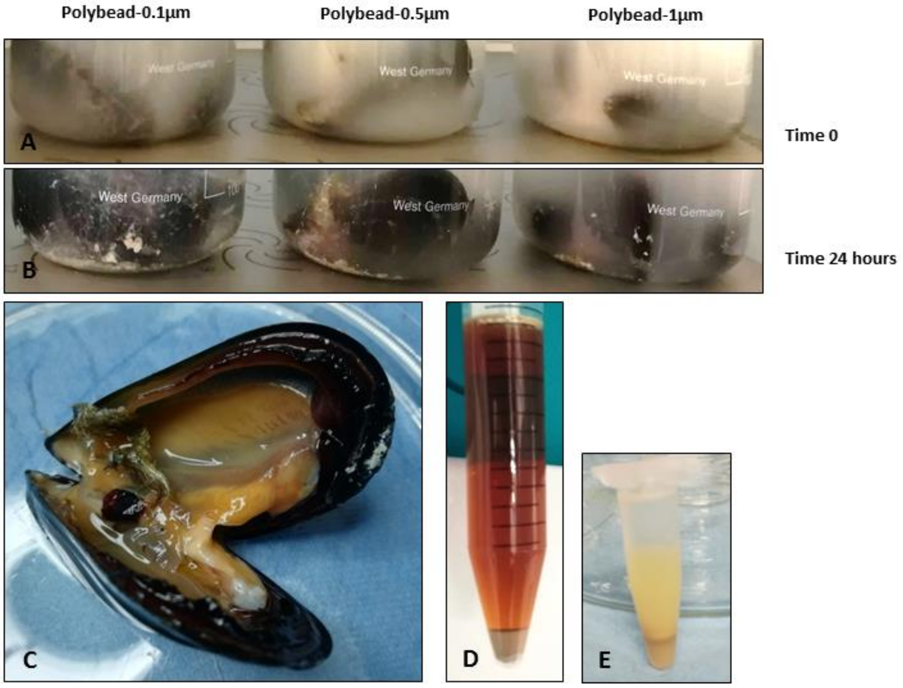

Figure SI1. Exposure of mussels to pNP of 0.1-0.5 and 1 µm diameter size. Time 0 (A); 24 hours of exposure (B); mussels exposed to pNP 500 nm diameter size at the end of exposure (C); digested mussel (D); one step of pellet D purification (E);

Table SI2. Elemental composition of the intestine of the mussel as measured by EDX

**Element (A)**	**Elemental Comp. (%)**
	Homogenized mussel intestine
C (12)	0.52
N (14)	0.03
O (16)	0.36
Na (23)	0.02
K (39)	< 0.01
S (32)	< 0.01
Cl	< 0.01

P	< 0.01
Others (Mg, Ca)	< 0.01

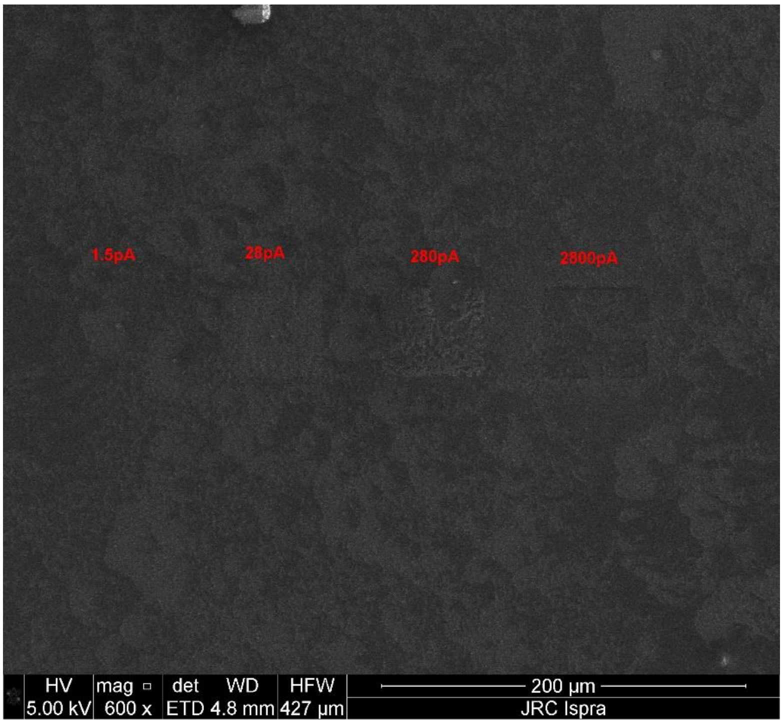

Figure SI2. SEM image of the monolayer of PS particles (1000 nm) irradiated with Ga^+ ions at 30KeV at different current of ions (determining different doses). The lowest dose is used in the experiments to clean the residual mussel matrix without affecting the PS structure.

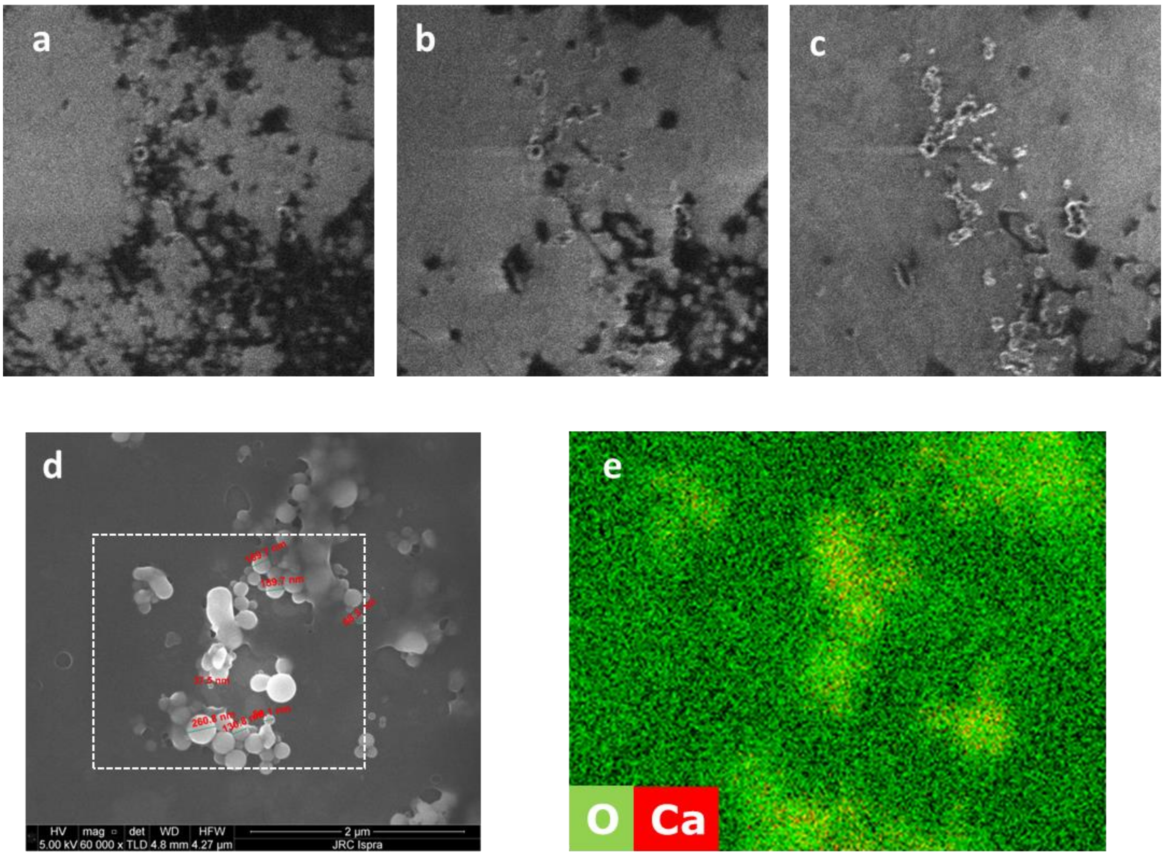

Figure SI3. SEM image of the mussels matrix irradiated with Ga^+ ions at 30KeV (dose = $6x10^{13}$ ions/cm^2) at (a) 30 sec (b) 60 sec (c) 90 sec. (d) High resolution SEM image of the round objects found after cleaning and (e) EDS map of the inset in (d) for Oxygen and Calcium.
